# Cytotoxic and Antioxidant Effects of Antimalarial Herbal Mixtures

**DOI:** 10.1155/2020/8645691

**Published:** 2020-02-10

**Authors:** Isaac Dadzie, Shaibu Adams Avorgbedo, Regina Appiah-Opong, Obed Cudjoe

**Affiliations:** ^1^Department of Medical Laboratory Science, School of Allied Health Sciences, University of Cape Coast, Cape Coast, Ghana; ^2^Department of Clinical Pathology, Noguchi Memorial Institute for Medical Research, University of Ghana, Legon, Ghana; ^3^Department of Microbiology and Immunology, School of Medical Sciences, University of Cape Coast, Cape Coast, Ghana

## Abstract

Many developing countries depend on herbal mixtures as the first line and cost-effective therapy for malaria. These mixtures with such curative tendencies may also be a source of toxicity to host cells. On the other hand, these mixtures may have anticancer potential activity characterized by cytotoxicity to cancer cells. The aim of the study was to determine the cytotoxic and antioxidant effects of five different antimalarial herbal mixtures. Five antimalarial herbal mixtures commonly used in Ghana (coded as STF, SMH, SMM, SGM, and STT) were purchased and freeze-dried. The dried samples were tested on human acute T-cell leukemia (Jurkat) and breast adenocarcinoma (MCF-7) cell lines. Cytotoxicity was assessed using the tetrazolium-based colorimetric (MTT) assay while antioxidant activity was determined using DPPH free-radical scavenging assay. Among the mixtures, SMM and SGM exhibited the strongest cytotoxicity towards Jurkat cells (IC_50_ values 59.17 *μ*g/ml and 49.57 *μ*g/ml, respectively), whereas STT showed the weakest cytotoxicity (IC_50_ = 244.94 *μ*g/ml). Cytotoxic effect of SMM was also strongest towards MCF-7 cells whilst the least cytotoxic sample was SGM (IC_50_ > 1000 *μ*g/ml). SMM had the highest antioxidant percentage (EC_50_ = 1.05 mg/ml). The increasing order of antioxidant percentage among the five herbal mixtures is SMM > SMH > STT > STF > SGM. The herbal mixtures may be potential sources of toxic agents to host cells. Therefore, further toxicity studies must be performed to safeguard health of the public. Interestingly, cytotoxicities exhibited by SMM and SGM suggest the presence of anticancer constituents in them which warrant further studies.

## 1. Introduction

Numerous strategies have been adopted to control the incidence of malaria; however, an estimated 216 million cases of malaria were recorded worldwide in 2017, with 445,000 deaths. Approximately 90% of all malaria deaths occur in Africa [1]. The resistance of the *Plasmodium* parasite to the available antimalarial drugs is a major challenge to the control of malaria [2]. Previously, chloroquine was the drug of choice in Ghana and other endemic African countries for treatment of uncomplicated malaria caused by *P. falciparum*, the parasite responsible for most malaria cases. Artemisinin combination therapies (ACTs) are currently the drugs of choice for the treatment of uncomplicated malaria in Ghana. The emergence of drug-resistant strains therefore necessitates intensive search for new drugs [3].

With the increasing levels of drug resistance and the high cost of treatment with conventional antimicrobial drugs, herbal mixtures readily serve as the most assessable and affordable means of treatment for many illnesses in many parts of the world [4, 5]. The majority of the population in many developing countries, where malaria is endemic, depends on herbal mixtures as therapy for malaria [6, 7]. Medicinal plants, such as *Uvaria chamae*, *Strophanthus hispidus*, *Psidium guajava*, *Cassia abbreviata*, *Aristolochia albida*, *Annona muricata*, *Mangifera indica*, and *Azadirachta indica* have been documented and are used in most African countries to treat malaria and other ailments, either alone or in combination with other plant medicines [6, 8–10].

The preference for herbal preparation has been partly informed by the fact that these herbs are of natural origin and are therefore considered safe to use as compared to the synthetic or the orthodox drugs. However, not all of such natural products are safe. Herbal remedies associated with adverse effects have been reported and continue to engage the attention of researchers [11–14]. There are so many herbal mixtures available and sold in Ghana for treatment of malaria. However, safety profile of the majority of these antimalarial mixtures has not yet been scientifically investigated. Hence, this study sought to determine the cytotoxic effect of five different commonly used antimalarial herbal mixtures sold on the Ghanaian market on cultured human cell lines.

## 2. Materials and Methods

Fetal bovine serum (FBS), RPMI culture medium, penicillin-streptomycin, and 3-(4,5-dimethylthiazol-2-yl)-2,5-diphenyltetrazolium bromide (MTT) were obtained from Sigma Chemical Company (St. Louis, MO, USA). Human acute T-cell leukemia (Jurkat) and breast adenocarcinoma (MCF-7) cell lines were obtained from RIKEN BioResource Center Cell Bank (Japan). All other reagents and chemicals used for the work were of analytical grade and obtained from standard suppliers.

### 2.1. Sample Preparation

Five (5) commonly used herbal antimalarial mixtures from different manufacturers were purchased from the market in Accra. These samples were coded (as STF, SMH, SMM, SGM, and STT), frozen at −20°C and freeze-dried.

### 2.2. Cell Culture

Jurkat cells were cultured in RPMI medium supplemented with 10% FBS and 1% penicillin-streptomycin, whilst MCF-7 cells were cultured in DMEM with the same supplements. The cells were maintained in a humidified incubator with 5% CO_2_ at 37°C and subcultured when they were about 80% confluent.

### 2.3. Cell Viability (MTT) Assay

Determination of cytotoxicity of the herbal extracts was carried out *in vitro* using the MTT assay as described in [15, 16]. Five different concentrations (62.5 to 1000 *μ*g/ml) of each of the herbal mixtures were prepared by serial dilution. Hundred microliters (100 *μ*l) cell suspension was added into each well in a 96-well microtitre plate, and 10 *μ*l of extract dilution was added. Triplicate experiments were performed. Curcumin was used as positive control. Negative control (vehicle, water) and extract control experiments were also set up. The plates were incubated in a humidified incubator with 5% CO_2_ at 37°C for 72 h. Twenty microliters (20 *μ*l) of 2.5 mg/ml MTT solution (in PBS) was added to each well of the plates, and incubation was continued for 4 h. After 4 hours, 150 *μ*l of acidified isopropanol was added to each well, and the plates were incubated in the dark at room temperature overnight. The MTT assay spectrophotometrically measures the purple-coloured formazan resulting from the reduction of the yellow tetrazolium salt by metabolically active cells. Absorbance was read at the wavelength of 570 nm using a microplate reader (Tecan Infinite M200, Austria). The effect of the herbal mixtures on cell viability was calculated using the following formula:

(1) $$\begin{array}{c} \text{viability}\left(\text{\%}\right)=\frac{A_{0}-A_{1}}{A_{0}}-B\times 100, \end{array}$$

where *A*_0_ is the mean absorbance of wells with untreated cells (vehicle), *A*_1_ is the absorbance of test wells, and *B* is the absorbance of blank wells (extract control, cell free). The concentration of the test sample that resulted in 50% decrease in the cell number, i.e., 50% inhibitory concentration (IC_50_), as compared with that of the control cultures (untreated cells) was then determined.

### 2.4. Determination of Antioxidant Activity: DPPH Assay

The scavenging activities of the powdered herbal mixtures on the stable free-radical DPPH (2,2-diphenyl-1-picryl-hydrazyl-hydrate) were assayed according to a method described, with slight modification [17, 18]. Serial dilutions of each sample were prepared (0.027–20 mg/ml). Each reaction mixture comprised 100 *μ*l of 0.5 mM DPPH solution (in methanol) and 100 *μ*l of the sample in 96-well plates. Butylated hydroxytoluene (BHT) was used as positive control. Triplicate experiments were performed. The plates were incubated for 20 min at room temperature in the dark, and the absorbance was read using a microplate reader (Tecan Infinite M200, Austria) at 517 nm. The percentage of inhibition was calculated using the equation:

(2) $$\begin{array}{c} \text{inhibition}\left(\text{\%}\right)=\frac{A_{0}-A_{1}}{A_{0}}\times 100, \end{array}$$

where *A*_0_ is the absorbance of the control and *A*_*i*_ is the absorbance of the samples. EC_50_, which is the concentration where 50% of the free-radical activity of DPPH is quenched, was extrapolated from a graph of percent antioxidant activity versus sample concentration [18].

## 3. Results

### 3.1. Cytotoxicity

**Figure 1:**
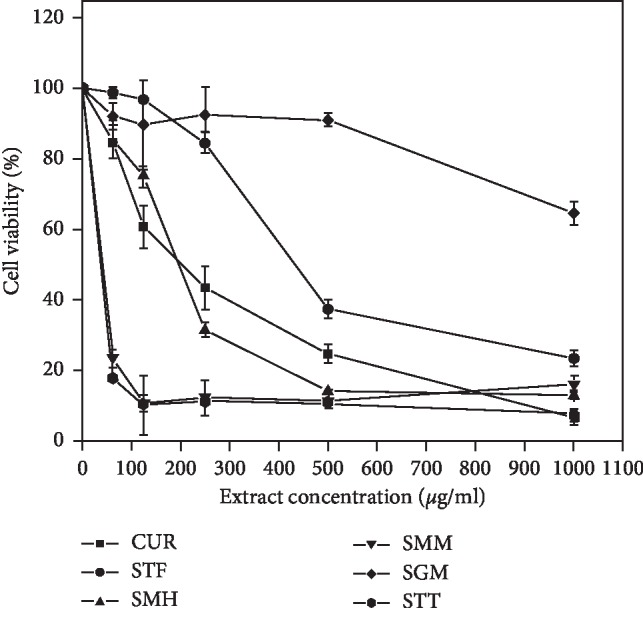
Cytotoxic effects of the herbal mixtures on Jurkat leukemia cells. Each plotted point represents the mean of three independent experiments, and the bars are standard deviations.

**Figure 2:**
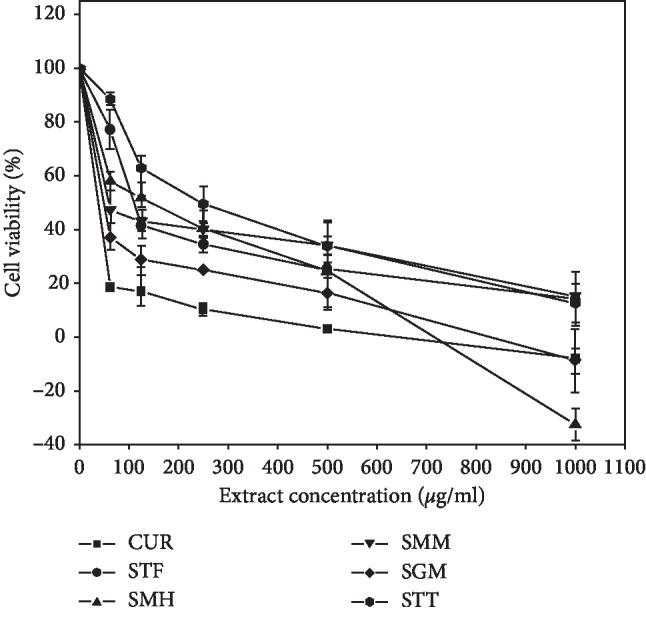
Cytotoxic effects of the herbal mixtures on MCF-7 breast cancer cells. Each plotted point represents the mean of three independent experiments, and the bars are standard deviations.

For evaluation of cytotoxicity, the cells were exposed to increasing concentrations of the herbal extracts with curcumin serving as positive control. Figures 1 and 2 show the dose-dependent effect of the herbal mixtures on Jurkat cell lines, with their corresponding IC_50_ values (Table 1). The antimalarial STT showed the least cytotoxic effect towards Jurkat cells with an IC_50_ value of 244.92 *μ*g/ml, whilst SGM gave the strongest cytotoxic effects (IC_50_ = 49.57 mg/ml) on the cell lines. On the other hand, among the herbal mixtures, SGM showed the least cytotoxicity towards MCF-7 cell lines (IC_50_ > 1000 mg/ml), whereas SMM gave the strongest activity (IC_50_ = 40.82 mg/ml) on the cells. The herbal mixtures STF and SMH exhibited weak cytotoxic activities towards both human cell lines (IC_50_ > 100 *μ*g/ml). Curcumin, which was used as the positive control exhibited a higher cytotoxicity against both cell lines as expected, with an IC_50_ value of 3.84 *μ*g/ml and 7.45 *μ*g/ml for Jurkat and MCF-7 cells, respectively.

### 3.2. Antioxidant Activity

Protection against oxidative damage is one of the most widely described attributes of plant extracts and relates to their radical scavenging activity. The scavenging properties of the herbal mixtures were evaluated by the DPPH radical scavenging assay. The EC_50_ values are a parameter widely used to measure antioxidant activity [19]. The lower the EC_50_ value, the higher the antioxidant activity.  Table 2 shows the EC_50_ values of the herbal mixtures and BHT, which was used as positive control. Among the mixtures, SMM gave the lowest EC_50_ value (1.05 mg/ml) which indicates the highest antioxidant activity. This was followed by SMH and STT which gave similar activities (EC_50_ values of 1.45 mg/ml and 1.65 mg/ml, respectively). The highest EC_50_ value was found for SGM.

## 4. Discussion

Traditional herbal medicines have been used to treat malaria for years, and they are the source of major groups (artemisinin and quinine derivatives) of potent modern antimalarial drugs [20]. Herbal preparations may achieve the desired curative purpose when administered but may also contain other ingredients that may have toxic effects on human. We assessed the cytotoxic effects of five known potent antimalarial herbal mixtures on human cell lines Jurkat and MCF-7. Antioxidant activities of the mixtures were also assessed to determine the potential of the products to protect the body against oxidative stress. The herbal mixtures that were considered in this study are commonly used mixtures claimed to be very effective antiplasmodial agents.

Results obtained show that the five antimalarial herbal mixtures tested had varying cytotoxic activities toward the human cell lines compared to the positive control (curcumin), which was highly cytotoxic to both cells. Possibly, the cytotoxic effect of individual components has been diluted out in the mixture. Alternatively, the observed cytotoxicity could be an additive or synergistic effect from the various components of the mixtures. *In vitro* analysis of potential toxic, mutagenic, and carcinogenic effects of herbal medicines has been established on both normal and cancer cells by many scientific studies [21–25]. These medicines have been reported to have therapeutic synergistic effect or may also be antagonistic for the side effects observed [26, 27]. Assessment of the effects of other commercial herbal preparations has been done in South Africa, where 6 commonly used preparations were tested for their effects on isolated human platelets [28]. Similar studies by Mothibe et al. on a herbal body-healing mixture observed variable stimulatory and inhibitory effects on human neutrophils [29].

The inhibitory effects of a herbal mixture in general have been attributed to the total activity of the crude extract, rather than that of a single major component of the mixtures [30], although this may not always be the case. Cancer cells are frequently used for cytotoxicity studies because of the rapid growth rate compared to normal cells, thus facilitating quick acquisition of *in vitro* data on test samples. More so, whatever harms cancer cells will also usually harm normal healthy cells in the body.

Figures 1 and 2 show the inhibitory effect of the herbal mixtures on the two cell lines. In all instances, the percentage cell viability decreased in a dose-dependent manner with increasing concentrations of the mixture. It could also be seen that toxic effect of mixtures on cells varied depending on the cell line used. Although both SMH and SMM were produced from the same plant component (*Cryptolepis sanguinolenta*) (Table 3), they showed different degrees of cytotoxicity on the cells tested. This difference may possibly be due to different extraction methods employed by the two different manufacturers. The activities may also partly be due to the fact that these two mixtures have other plant components with cytotoxic effect which have not been stated on their respective labels.

Four out of the five herbal mixtures considered in this study were either made from *Cryptolepis sanguinolenta* or had the plant as a component of the mixture. Indeed, antimalarial activity of extracts of this plant has been long established in Africa [31–33]. Cytotoxicity of aqueous extract of *Cryptolepis sanguinolenta* on different cell lines has also been reported by Ansah and Goodman [34]. Except for SMM which was cytotoxic even at a lower concentration, cytotoxicity increased with increasing concentrations of the mixtures. In most rural communities, extract of these plants may be the only alternative for the treatment of malaria. This raises the concern on how much of these mixtures one needs to consume to achieve the desired curative purpose without experiencing significant toxic effect.

Plant-based medicines generally contain a significant amount of phyto-antioxidants which prevent oxidative damage to the hosts such as that which is caused by the *Plasmodium* parasite from malaria infection. We also tested whether these mixtures have antioxidant effects. All the herbal mixtures had high antioxidant activities compared to the positive control, with SMM recording the strongest activity (EC_50_ = 1.05 mg/ml). The importance of antioxidant activity is to slow down or prevent the oxidative damage to the host cells caused by oxidation reactions that produce free radicals [35, 36]. Malaria infection induces the generation of hydroxyl radicals in the liver, which can lead to the induction of oxidative stress and apoptosis [37]. These stresses are managed through various cellular enzymatic and nonenzymatic systems enhanced by certain compounds, most of which are found in plants. Thus, the curative ability of the herbal mixtures, as testified by consumers, could partly be attributed to the relatively high antioxidant activity of these mixtures.

It is worth noting that SMM had the highest percentage antioxidant activity among the five herbal medicines as well as have the highest cytotoxic effect on the two cancer cells that we used. The high antioxidant activity of SMM, coupled with its strong effect on cancer cells, could be explored in the treatment of cancer. The active components of this herbal preparation (*Cryptolepis sanguinolenta*) could be a subject for further investigation as far as treatment of cancer is concerned.

In conclusion, although these herbal mixtures are claimed to be potent antimalarial agents they could be potentially toxic to host cells. Therefore, comprehensive toxicity studies are warranted to safeguard public health. Also, close attention must be paid to the dosage and the patient's health history since certain diseases could be secondary factors in the generation of toxicities in the presence of these herbal mixtures.

## Figures and Tables

**Table 1:** *In vitro* cytotoxic effects of the herbal mixtures on human cells.

Herbal mixtures	IC50 values of JURKAT cells and MCF-7 cells (*μ*g/ml)	
STF	110.6 ± 8.46	432.78 ± 5.77
SMH	143.99 ± 13.22	196.55 ± 2.88
SMM	59.17 ± 11.54	40.82 ± 1.52
SGM	49.57 ± 4.10	1000 ± 4.35
STT	244.94 ± 10.41	97.95 ± 1.15
CUR	3.80 ± 0.15	7.45 ± 0.11

The values represent the mean of three independent experiments. CUR: curcumin, positive control.

**Table 2:** Antioxidant activity of the herbal mixtures.

Herbal mixtures	EC50 (mg/ml)
STF	2.69 ± 0.35
SMH	1.45 ± 0.23
SMM	1.05 ± 0.21
SGM	3.58 ± 0.31
STT	1.65 ± 0.38
BHT	0.23 ± 0.02

BHT, butylated hydroxytoluene, a known potent antioxidant was used as positive control.

**Table 3:** Herbal antimalarial mixtures and their plant components.

Herbal mixtures	Mass of dried extract (mg)^*∗*^	Plant component
STF	0.34	*Azadirachta indica*, *Alstonia boonei*
SMH	0.69	*Cryptolepis sanguinolenta*
SMM	0.17	*Cryptolepis sanguinolenta*
SGM	0.07	*Cryptolepis sanguinolenta*, *Morinda lucida*, *Nauclea latifolia*
STT	0.03	*Carapa procera*, *Cryptolepis sanguinolenta*

^*∗*^Mass of the extract obtained after freeze drying 30 ml of the herbal mixtures.

## Data Availability

The data used to support the findings of this study are available from the corresponding author upon request.
